# Case Report: Single-Cell Transcriptomic Analysis of an Anaplastic Oligodendroglioma Post Immunotherapy

**DOI:** 10.3389/fonc.2020.601452

**Published:** 2021-01-14

**Authors:** Guangyang Yu, Madison K. Butler, Abdalla Abdelmaksoud, Ying Pang, Yu-Ting Su, Zachary Rae, Kimia Dadkhah, Michael C. Kelly, Young K. Song, Jun S. Wei, Masaki Terabe, Ramya Atony, Kelly Mentges, Brett J. Theeler, Marta Penas-Prado, John Butman, Kevin Camphausen, Kareem A. Zaghloul, Edjah Nduom, Martha Quezado, Kenneth Aldape, Terri S. Armstrong, Mark R. Gilbert, James L. Gulley, Javed Khan, Jing Wu

**Affiliations:** ^1^Neuro-Oncology Branch, Center for Cancer Research, National Cancer Institute, National Institutes of Health, Bethesda, MD, United States; ^2^Genetics Branch, Center for Cancer Research, National Cancer Institute, National Institutes of Health, Bethesda, MD, United States; ^3^Single Cell Analysis Facility, Center for Cancer Research, National Institutes of Health, Bethesda, MD, United States; ^4^Diagnostic Radiology Department, The Clinical Center of the National Institutes of Health, Bethesda, MD, United States; ^5^Radiation Oncology Branch, National Cancer Institute, National Institutes of Health, Bethesda, MD, United States; ^6^Surgical Neurology Branch, National Institute of Neurological Disorders and Stroke, National Institutes of Health, Bethesda, MD, United States; ^7^Laboratory of Pathology, Center for Cancer Research, National Cancer Institute, National Institutes of Health, Bethesda, MD, United States; ^8^Genitourinary Malignancies Branch, National Cancer Institute, National Institutes of Health, Bethesda, MD, United States

**Keywords:** glioma, immunotherapy, tumor microenvironment, pseudo-progression, single-cell RNA sequencing

## Abstract

Glioma is the most common primary malignant brain tumor with a poor prognosis. Immune checkpoint inhibitors have been of great interest in investigation of glioma treatments. Here, we report single-cell transcriptomic analyses of two tumor areas from an oligodendroglioma taken from a patient who had multiple tumor recurrences, following several chemotherapies and radiation treatments. The patient subsequently received nivolumab and was considered have disease progression based on conventional diagnostic imaging after two cycles of treatment. He underwent a debulking surgical resection and pathological diagnosis was recurrent disease. During the surgery, tumor tissues were also collected from the enhancing and non-enhancing areas for a scRNAseq analysis to investigate the tumor microenvironment of these radiographically divergent areas. The scRNAseq analysis reveals a plethora of immune cells, suggesting that the increased mass observed on MRI may be partially a result of immune cell infiltration. The patient continued to receive immunotherapy after a short course of palliative radiation and remained free of disease progression for at least 12 months after the last surgery, suggesting a sustained response to immunotherapy. The scRNAseq analysis indicated that the radiological progression was in large part due to immune cell infiltrate and continued immunotherapy led to a positive clinical outcome in a patient who would have otherwise been admitted to hospice care with halting of immunotherapy. Our study demonstrates the potential of scRNAseq analyses in understanding the tumor microenvironment, which may assist the clinical decision-making process for challenging glioma cases following immunotherapy.

## Introduction

Malignant gliomas, which represent about 80% of primary malignant brain tumors, remain as an incurable disease, due to many treatment challenges unique to brain tumors (1–3). With the increasing evidence of the existence of infiltrating immune cells and immune surveillance in the brain, immunotherapy has become an interest in brain tumors (4, 5). There have been positive outcomes with immunotherapy in multiple cancer types, leading to FDA approval of nivolumab, a monoclonal antibody targeting PD-1, and ipilimumab, a monoclonal antibody binding to CTLA-4 for treatment of melanoma and non-small cell lung cancer (6–8). There is increasing interest in exploring immunotherapy for brain tumors. Safety profiles have been established in patients with newly diagnosed glioblastoma treated with nivolumab and ipilimumab (9).

The management of glioma patients during immunotherapy remains a challenge as there are no clear radiological features to distinguish response from progression using the conventional magnetic resonance imaging (MRI) (10). MRI is the most commonly used approach for assessment of disease progression and relies on contrast enhancement to distinguish malignant transformation which usually results in breakdown of the Blood Brain Barrier (BBB) (11). However, radiographic changes post immunotherapy such as increased contrast enhancement, enlargement of existing lesions, and/or appearance of new lesions that are not due to tumor progression have been observed and classified as pseudo-progression (12). Pseudo-progression has been reported in glioma patients receiving concurrent chemoradiation and immunotherapy (13–16). Radiographic changes after immunotherapy may occur for multiple reasons aside from disease progression. Although early radiographic changes may reflect true disease progression before the immune response has initiated (16), an increase in enhancing signals on MRI may represent an inflammatory response, resulting from infiltration of tumor-infiltrating immune cells into the tumor (17). Pseudo-progression may occur with or without observation of clinical deterioration, which further confounds the interpretation of radiographic findings (18). Understanding the impact of glioma immunotherapy at the cellular level will benefit the clinical decision-making.

Here, we present a patient with a recurrent anaplastic oligodendroglioma (AO), who showed a prolonged response to immune therapy despite disease progression by the conventional MRI with pathological confirmation. It highlights the challenge of interpreting radiographic progression in gliomas and demonstrates the potential of additional molecular testing to assist the clinical decision-making for improving treatment response.

## Case Presentation

**Figure 1:**
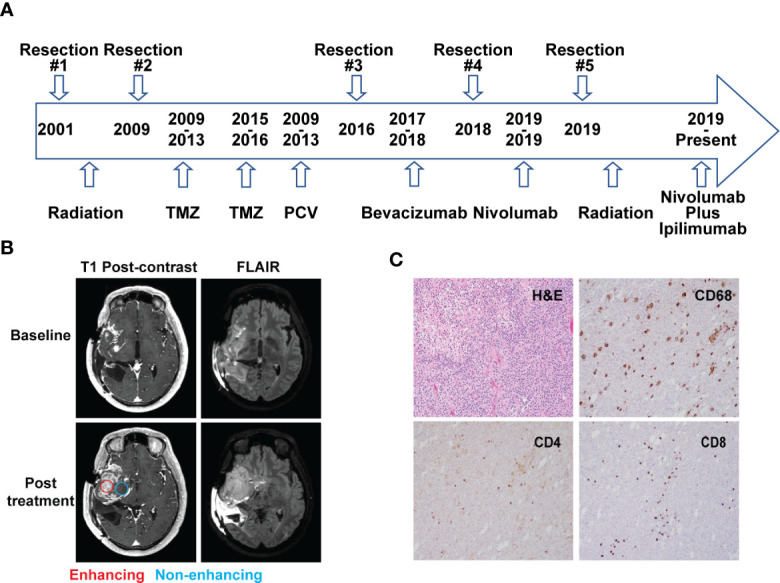
Case presentation. **(A)** Timeline of disease diagnosis and treatment. **(B)** Magnetic resonance imaging (MRI) obtained at baseline (top panel) and after 2 cycles of nivolumab treatment (bottom panel). T1 post-contrast and FLAIR sequences are shown in the left and right column, respectively. Red and blue circles indicate the location of tissue acquisition from the enhancing (red) and non-enhancing (blue) regions. **(C)** Immunohistochemistry analysis showing H&E staining and reactivity of immune cell markers CD68, CD4, and CD8 (10× magnification) from the resected tissue following nivolumab treatment.

A 48-year-old man who initially presented with seizures was subsequently diagnosed with AO, WHO grade III nineteen years prior to this study. As illustrated in **Figure 1A**, he received radiation therapy (RT) after the initial surgery to remove the tumor. Eight years later, he had a second resection followed by two years of temozolomide (TMZ) treatment. The patient then received five cycles of procarbazine, lomustine and vincristine (PCV) treatment due to the concern of disease progression. Four years prior to the current presentation, he underwent a third surgical resection after disease progression and was treated with bevacizumab for a year until the disease was found to recur. The patient underwent a fourth resection two years prior to presentation to the National Institutes of Health (NIH) for further evaluation. He was enrolled in a clinical trial (NCT03718767) and started nivolumab treatment. An MRI scan after two cycles of treatment (nivolumab 240 mg intravenously, every two weeks on a 28 day-cycle) showed that the enhancing lesions in the right insular region increased in size significantly, while the fluid-attenuated inversion recovery (FLAIR) signal in the right frontal and temporal lobe remained largely unchanged from the baseline, suggesting worsening high grade glioma. (**Figure 1B**). A surgical debulking was indicated due to potentially life-threatening mass effect and a craniotomy with tumor resection was performed. The pathologic exam showed a mixture of tumor cells and immune cell infiltration which suggested treatment effects (**Figure 1C**). The final diagnosis was recurrent AO. Post-surgically, the patient’s overall condition became worse but without focal neurological deficit. The patient was recommended comfort care with a short course of palliative radiation to the tumor resection bed.

## Diagnostic Assessment

### Immunohistological Exam

A hematoxylin and eosin (H&E) stained tumor slide showed a neoplastic process with oligodendroglia phenotype including cells with round nuclei. High grade features including marked cellularity, cell atypia, mitoses, vascular proliferation and pseudopalisading necrosis were present. Additional immunohistochemistry (IHC) exam of immune cells revealed the presence of CD4+ and CD8+ T cell infiltration. CD68+ cells, representing macrophage and microglia cell populations, were also identified on the tumor slides (**Figure 1C**).

### Single-Cell RNA Sequencing

To better understand the tumor microenvironment, particularly the immune cell distribution in radiographically different areas following immunotherapy, the samples were collected from both enhancing and non-enhancing lesions during the tumor resection (lower left, **Figure 1B**), and processed for single-cell RNA sequencing (scRNAseq) analysis.

#### Cell Type Distribution in Tumor Tissue

**Figure 2:**
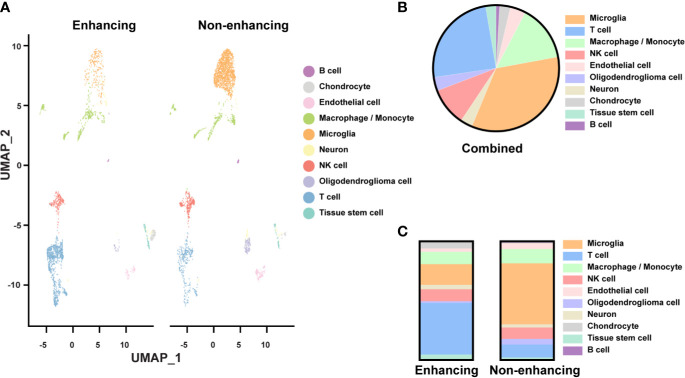
Single-cell analysis of resected tissue from enhancing and non-enhancing lesions **(A)** Uniform Manifold Approximation and Projection (UMAP) representation of ten identified cell types in enhancing and non-enhancing samples from the resected tissue. **(B)** Percentages of each cell type of all cell populations. **(C)** Percentages of each cell type of the enhancing and non-enhancing samples.

We identified ten major cell types based on their gene expression profiles (**Figure 2A**). Though the tissues were expected to be composed of mostly tumor, only 3.5% of the cells were classified as oligodendroglioma cells (**Figure 2B**). Most of the cell clusters were identified as immune cell types, including microglia (34.2%), macrophages/monocytes (14.4%), NK cells (9.8%), T cells (24.6%), and a small amount of B cells (0.9%) (**Figure 2B**). Other non-tumor cells included endothelial cells (4%), chondrocytes (2.8%), tissue stem cells (2.7%), and neurons (3.1%). Thus, based on the single-cell analysis, almost 84% of the cells analyzed were immune cells and less than 5% of oligodendroglioma cells. The low percentage of tumor cells in the single-cell population determined by the scRNAseq seems to be lower than that detected by the H&E staining on tumor tissue slides. This difference may be due to tissue sampling or single-cell processing, including dead cell removal. Here, we focused on the immune microenvironment.

Notably, cell type distribution was quite different in enhancing versus non-enhancing lesions. Over half (51.5%) of the cells in non-enhancing lesion were composed of microglia compared to only 10.4% of the cells in enhancing tissue (**Figure 2C**). There was a slightly higher percentage of macrophages/monocytes in the enhancing sample (17.6%) compared with non-enhancing sample (12.1%). Interestingly, 43.6% of the cells from enhancing lesion were composed of T cells compared to 10.9% of the cells from non-enhancing region. The percentages of NK cells within each sample were similar.

#### Functional Status of Immune Cells

**Figure 3:**
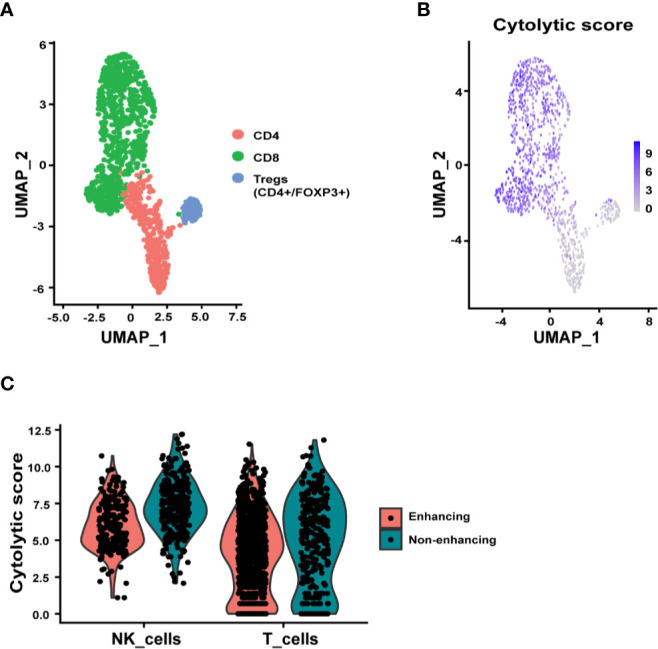
Subclustering analysis of T and NK cells. **(A)** UMAP plot showing three distinct T cell subpopulations, CD4+, CD8+, and Tregs (CD4+/FOXP3+). **(B)** UMAP representation of cytolytic score of T cells. **(C)** Violin plots summarizing the cytolytic score of NK cells and T cells from enhancing and non-enhancing samples. The average cytolytic scores of T cells were 1.87 in enhancing and 2.50 in non-enhancing lesions (Wilcoxon test p = 3.084 x 10-6), and the average cytolytic scores of NK cells were 3.06 in enhancing and 4.66 in non-enhancing (Wilcoxon test p = 4.136 x 10-14).

Since T cells, NK cells, microglia, and macrophages/monocytes were the most represented immune cell types in the tumor tissue, we further examined the relevant functions of these immune cells. Sub-clustering analysis of the T cells identified CD8+, CD4+, and regulatory T cell (Treg, CD4+/FOXP3+) subpopulations (**Figure 3A**). The CD8+ T cells, as expected, expressed the highest cytolytic score of T cells while the Tregs expressing at the lowest cytolytic score (**Figure 3B**). Interestingly there were many more T cells in the enhancing region (**Figure 2A**). When the cytolytic score per cell was calculated, a fold-change of 1.33 in non-enhancing lesion versus enhancing lesion was found (Wilcoxon test p = 3.084 x 10-6) (**Figure 3C**). Although we discovered that T cells in non-enhancing lesion expressed higher levels of *PRF1*, *GZMM*, *GZMH*, and *GZMB* when compared to that in the enhancing lesion, the significance of this is unknown and more studies are needed to define this in the context of immune therapy (**Figure S1**). Like CD8+ T cells, NK cells in the non-enhancing region expressed a higher cytolytic score compared to that of enhancing region (**Figure 3C**, fold change 1.52; Wilcoxon test, p = 4.136 x 10-14), though the percentages of NK cells within each sample were similar. By analyzing differential gene expression (DGE), we found that *SH2D2A*, encoding T cell Specific Adapter protein (TSAd), which mediates the activation of T cells, increased in the T cells of non-enhancing lesion (**Figure S2**) (19). *GNLY*, which encodes granulysin, a protein presents in the cytotoxic granules that are released by the activated CTLs, was found to be upregulated in T cells from non-enhancing tumor area (**Figure S2**) (20). *NFKB1*, which regulates the maturation and effector function of NK cells, was the only gene upregulated in NK cells of the enhancing lesion (**Figure S3**) (21). These findings may not be applicable to all glioma cases. However, the DGE analysis at the single-cell level may help to explain the role of T/NK cells in the immune tumor microenvironment.

**Figure 4:**
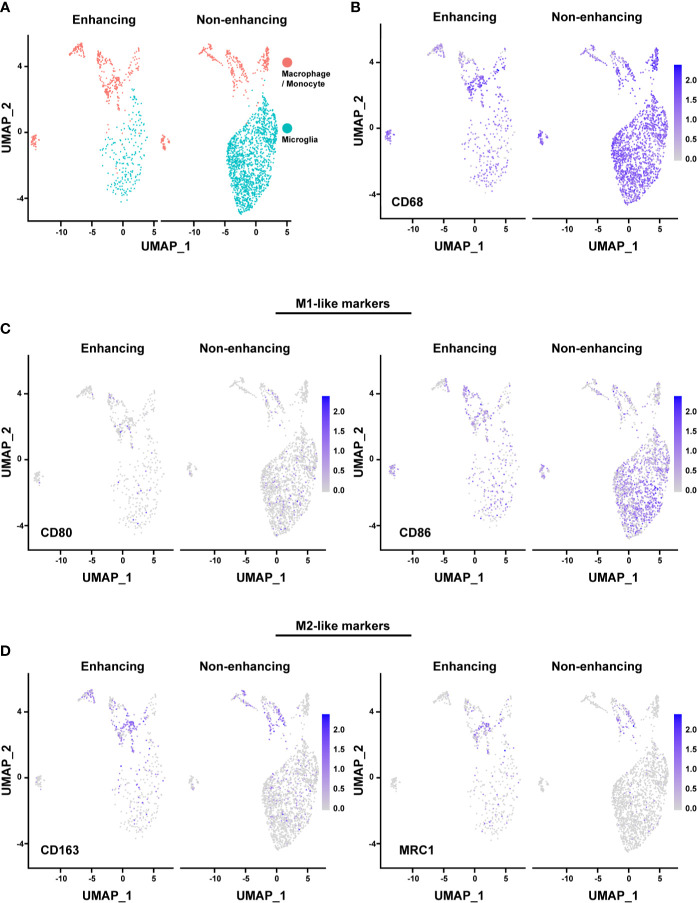
Analysis of macrophages/monocytes and microglia. **(A)** UMAP showing the distinct populations of macrophage/monocyte and microglia in enhancing and nonenhancing lesion. **(B)** CD68+ cells in the macrophage/monocyte and microglia populations in enhancing and non-enhancing lesion. **(C)** M1-like markers expression distribution in macrophage/monocyte and microglia populations in enhancing and non-enhancing lesion. CD80 (left) and CD86 (right). **(D)** M2-like markers expression distribution in macrophage/monocyte and microglia populations in enhancing and non-enhancing lesion. CD163 (left) and MRC1 (right).

The majority of the microglia were found in the non-enhancing lesion, while macrophages/monocytes have a similar distribution in both enhancing and non-enhancing lesion (**Figure 4A**). As expected, both cell clusters expressed CD68, consistent with the identity of the cell types (**Figures 4B**). We used the markers of CD80, CD86, CD163, and MRC1 (CD206) to further characterize the functional status of the subpopulations. The expression of the M1-like (pro-inflammatory) cell marker CD80 was minimal throughout, while the CD86+ cells were much more enriched in both non-enhancing and enhancing lesions (**Figure 4C**). The expression of CD86 was found in both microglia and macrophages/monocytes. The expression of M2-like (anti-inflammatory) markers was found more in macrophages/monocytes but marginally in microglia population, suggesting that microglia may have more proinflammatory phenotype than the macrophages/monocytes in this case. Both M1-like and M2-like markers were found to be expressed in macrophages/monocytes to a similar extent in the enhancing and non-enhancing regions (**Figure 4D**). The DGE analysis revealed that *RNASET2*, which mediates the M1-like polarization and suppresses M2-like polarization, was upregulated in microglia and macrophages/monocytes of the non-enhancing lesion (**Figure S2**). This finding suggests a potential role of *RNASET2* in an antitumor microenvironment after immune therapy (22, 23). Together, the scRNAseq data indicate that the tumor microenvironment in this case is enriched with pro-inflammatory/anti-tumoral microglia after anti-PD-1 treatment.

#### Therapeutic Intervention and Clinical Outcome

Overall, the results demonstrate the presence of proinflammatory microglia, macrophages/monocytes and CD8+ T cells with high cytolytic scores in the tumor microenvironment after a short course of anti-PD-1 therapy, suggesting an immune response. Encouraged by the evidence of the immune response, the patient was continued on immunotherapy rather than hospice care. After a short course of palliative radiation around the resection cavity, he went on protocol “Care of the Adult Oncology Patient, NCI” (NCT00923065) to receive ipilimumab and nivolumab. Ipilimumab was administered at 1mg/kg every four weeks in combination with nivolumab 3 mg/kg every two weeks for four cycles followed by nivolumab alone at 480 mg every four weeks for twelve cycles (each cycle is four weeks). Since the most recent surgery, he has remained radiographically and clinically stable for at least 12 months (**Figure S4**). Thus, the results of the scRNAseq analysis are supported by the prolonged durable response to immunotherapy in this patient.

## Discussion

Here we report a case of recurrent anaplastic oligodendroglioma with a durable response to repeated immunotherapy. The re-challenge with immune therapy was provided after the patient was confirmed with disease progression by conventional MRI and pathologic exam after a short course of immunotherapy. The results of single-cell analysis using the tumor samples collected during the debulking surgery provided insightful information about the tumor microenvironment in both enhancing and non-enhancing lesions, suggesting an immune response to the prior treatment with nivolumab. More importantly, the patient remained free of progression for at least 12 months on continued immunotherapy, consistent with the findings of functional and proinflammatory immune cells from the scRNAseq analysis.

Like other case reports, the major limitation of this study stems from the nature of case reports. The findings from any single case may not be applicable to all cases. However, this case report highlights the challenges of interpreting imaging changes in glioma patients receiving immunotherapy. This case establishes a feasibility of obtaining in-depth information of tumor microenvironment by using single-cell analysis in glioma patients. The findings illustrate the different pathophysiology in radiographically different areas. Such insights can help to understand the biology and possibly assist in clinical decision making.

Accurately interpreting radiographic changes is of immense importance in neuro-oncology clinical management, as treatment may be prematurely discontinued in responding patients if tumor progression is inaccurately defined by MRI exam. More importantly, it can delay the adequate treatment for such patients. Immunotherapy has added increasing difficulty to interpreting imaging responses, such as inflammatory responses which often induce more enhancing lesions on the conventional MRI, mimicking disease progression (16). In recognizing these challenges, the immunotherapy Response Assessment for Neuro-Oncology (iRANO) working group has proposed guidelines to address the uncertain radiographic changes that may occur after immunotherapy treatment (13). In this case report, we used scRNAseq to investigate the tumor microenvironment of enhancing and non-enhancing tissues within the same tumor. Different immune cell infiltration patterns were found between enhancing and non-enhancing tissues. The findings of a large amount of T cells in the enhancing lesion may explain those instances where the increased enhancing lesion were confirmed not to be caused by tumor progression. The enrichment of proinflammatory microglia in this case suggest that the change of non-enhancing lesion may need to be evaluated carefully when a clinical decision is needed. Overall, the scRNAseq may help us understand the tumor microenvironment in regions with radiographic differences, which may provide insights into disease processes and further assist in clinical decision-making.

Pathologic diagnosis remains the gold standard for tumor diagnosis. However, smaller biopsies may cause sampling bias. Even with enough amount of tissue, sampled staining results may contain evidence of both tumor cells and treatment effects, resulting in a “mixed” diagnosis, making the treatment decision challenging. While it is essential to have information regarding the presence or absence of tumor cells in a tissue specimen, it is also important to understand the extent of the disease. It becomes more relevant in the context of immunotherapy, where an overall tumor control is determined by a dynamic balance between tumor growth and immune modulation. Compared to the pathologic analysis and bulk RNA sequencing, scRNAseq provides a much higher resolution due to the platform’s ability to analyze the RNA expression at the single-cell level, rather than averaging expression measurements of all cell types in the bulk tissue (24). Other studies have used this technology to investigate the landscape of gliomas and the tumor microenvironment. In a study that analyzed tumor core and peritumor samples from four patients with glioblastoma, a scRNAseq study after immunotherapy revealed that on average, 36% of the cells were neoplastic and 46.3% were immune-related (25). In another study that performed single-cell sequencing of unsorted samples from eight high-grade gliomas, six were primarily composed of glioma cells with minimal presence of immune cells (26). However, two of the eight tumors were composed of a larger fraction of immune cells, one with 48% myeloid cells, 5% T cells, and 45% tumor cells and the other with 57% myeloid cells and 43% tumor cells. Furthermore, there is evidence suggesting that IDH-mutant tumors contain lower numbers of immune cell infiltrates compared with wild-type (27). In our study, we show that the IDH-mutant sample from our case contained ~84% of immune cells, suggesting an enhanced immune cell infiltration compared with the intrinsic immune cell profiles of gliomas in the literature. Though the tumor microenvironment profiles may vary between patients, the predominant immune cell group identified by scRNAseq in glioma is primarily microglia/macrophages (28). In addition to a large number of microglia/macrophages, particularly pro-inflammatory microglia, a large percentage of cytotoxic T and NK cells were also detected. It provides with further evidence that the enrichment of immune cell populations more likely results from the response to immunotherapy than the resident glioma-associated immune cells.

Although the findings are intriguing and may provide with some insights of the tumor microenvironment in the radiographic different tumor areas following immunotherapy, they are inconclusive in nature. Even though the scRNAseq finding of this case is supported by a durable clinical response, other factors may have contributed to this favorable treatment response, including the palliative radiation prior to re-challenging with immunotherapy. Another confounding factor includes the fact that the second immunotherapy treatment was initially a combined treatment of nivolumab and ipilimumab for four cycles, then followed by nivolumab, rather than nivolumab alone. A prospective controlled study is warranted to elucidate the treatment response. Other limitations of scRNAseq is that it may be noisier and more difficult to interpret than bulk RNA sequencing, and refined methods for sample collection, processing, and bioinformatic analysis must be standardized for a maximum reliability and reproducibility for clinical use in directing patient care.

In summary, our study established the feasibility of using the scRNAseq technology as a strategy to investigate the tumor microenvironment in the context of immunotherapy in gliomas. The potentials of scRNAseq analyses in assisting clinical decision-making in challenging case are demonstrated. A prospective evaluation of the correlation of scRNAseq with radiographic findings in glioma following immunotherapy may also be considered.

## Data Availability Statement

The raw data supporting the conclusions of this article will be made available by the authors, without undue reservation.

## Ethics Statement

The studies involving human participants were reviewed and approved by Institutional Review Board of National Institutes of Health. The patients/participants provided their written informed consent to participate in this study.

## Author Contributions

GY, MB, AA, MK, JK, and JW drafted the manuscript. GY, MB, YP, KD, AA, JW, MT, JW, and JK contributed to data analysis and interpretation. GY, MB, Y-TS, and ZR prepared single cells. MK, ZR, and Y-TS contributed to the sequencing and library preparation. RA, KZ, EN, JB, KC, JG, KM, BT, MP-P, TA, MG, and JW involved in patient care. MQ and KA performed pathologic diagnostic testing. All authors contributed to the article and approved the submitted version.

## Conflict of Interest

The authors declare that the research was conducted in the absence of any commercial or financial relationships that could be construed as a potential conflict of interest.

## References

[1] Ostrom QT Cioffi G Gittleman H Patil N Waite K Kruchko C CBTRUS statistical report: primary brain and other central nervous system tumors diagnosed in the United States in 2012–2016. Neuro Oncol (2019) 21(Supplement_5):v1–v100. 10.1093/neuonc/noz150 31675094 PMC6823730

[2] Kamran N Alghamri MS Nunez FJ Shah D Asad AS Candolfi M Current state and future prospects of immunotherapy for glioma. Immunotherapy (2018) 10(4):317–39. 10.2217/imt-2017-0122 PMC5810852 29421984

[3] Rajesh Y Pal I Banik P Chakraborty S Borkar SA Dey G Insights into molecular therapy of glioma: current challenges and next generation blueprint. Acta Pharmaco Sin (2017) 38(5):591–613. 10.1038/aps.2016.167 PMC5457688 28317871

[4] Chukwueke UN Wen P Use of the Response Assessment in Neuro-Oncology (RANO) criteria in clinical trials and clinical practice. Front Oncol (2019) 8:86(1):86. 10.3389/fonc.2018.00086 PMC6499019 30806082

[5] McGranahan T Therkelsen KE Ahmad S Nagpal S Current state of immunotherapy for treatment of glioblastoma. Curr Treat Options Onco (2019) 20(3):24. 10.1007/s11864-019-0619-4 PMC6394457 30790064

[6] Seidel JA Otsuka A Kabashima K Anti-PD-1 and anti-CTLA-4 therapies in cancer: mechanisms of action, efficacy, and limitations. Front Oncol (2018) 8:86. 10.3389/fonc.2018.00086 29644214 PMC5883082

[7] Hargadon KM Johnson CE Williams C Immune checkpoint blockade therapy for cancer: an overview of FDA-approved immune checkpoint inhibitors. Int Immunopharmacol (2018) 62:29–39. 10.1016/j.intimp.2018.06.001 29990692

[8] Kruger S Ilmer M Kobold S Cadilha BL Endres S Ormanns S Advances in cancer immunotherapy 2019–latest trends. J Exp Clin Cancer Res (2019) 38(1):1–11. 10.1186/s13046-019-1266-0 31217020 PMC6585101

[9] Sloan AE Gilbert MR Zhang P Aldape KD Wu J Rogers LR NRG BN002: Phase I study of checkpoint inhibitors anti-CTLA-4, anti-PD-1, the combination in patients with newly diagnosed glioblastoma. J Clin Oncol (2018) 36(15):2053–3. 10.1200/JCO.2018.36.15_suppl.2053

[10] Ranjan S Quezado M Garren N Boris L Siegel C Neto OLA Clinical decision making in the era of immunotherapy for high grade-glioma: report of four cases. BMC Cancer (2018) 18(1):239. 10.1186/s12885-018-4131-1 29490632 PMC5831705

[11] Huang RY Neagu MR Reardon DA Wen P Pitfalls in the neuroimaging of glioblastoma in the era of antiangiogenic and immuno/targeted therapy–detecting illusive disease, defining response. Front Neurol (2015) 6:33. 10.3389/fneur.2015.00033 25755649 PMC4337341

[12] Brandsma D Stalpers L Taal W Sminia P van den Bent M Clinical features, mechanisms, and management of pseudoprogression in malignant gliomas. Lancet Oncol (2008) 9(5):453–61. 10.1016/S1470-2045(08)70125-6 18452856

[13] Wen PY Chang SM Van den Bent MJ Vogelbaum MA Macdonald DR Lee E Response assessment in neuro-oncology clinical trials. J Clin Oncol (2017) 35(21):2439. 10.1200/JCO.2017.72.7511 28640707 PMC5516482

[14] Galldiks N Kocher M Langen K-J Pseudoprogression after glioma therapy: an update. Expert Rev Neurother (2017) 17(11):1109–15. 10.1080/14737175.2017.1375405 28862482

[15] Kucharczyk MJ Parpia S Whitton A Greenspoon JNJN-OP. Evaluation of pseudoprogression in patients with glioblastoma. Neurooncol Pract (2017) 4(2):120–34. 10.1093/nop/npw021 PMC6655498 31386017

[16] Okada H Weller M Huang R Finocchiaro G Gilbert MR Wick W Immunotherapy response assessment in neuro-oncology: a report of the RANO working group. Lancet Oncol (2015) 16(15):e534–e42. 10.1016/S1470-2045(15)00088-1 PMC4638131 26545842

[17] Yi L Ming H Yu S Ren B Yang XJG Ongoing evolution of response assessment in glioma: Where do we stand? Glioma (2018) 1(3):97. 10.4103/glioma.glioma_13_18

[18] Da Cruz LH Rodriguez I Domingues R Gasparetto E Sorensen A Pseudoprogression and pseudoresponse: imaging challenges in the assessment of posttreatment glioma. Am J Neuroradiol (2011) 32(11):1978–85. 10.3174/ajnr.A2397 PMC7964401 21393407

[19] Hem CD Ekornhol M Granum S Sundvold-Gjerstad V Spurkland A CD 6 and Linker of Activated T Cells are Potential Interaction Partners for T Cell-Specific Adaptor Protein. Scand J Immunol (2017) 85(2):104–12. 10.1111/sji.12513 27896837

[20] Sparrow E Bodman-Smith M Granulysin: The attractive side of a natural born killer. Immunol Lett (2020) 217:126–32. 10.1016/j.imlet.2019.11.005 31726187

[21] Lougaris V Patrizi O Baronio M Tabellini G Tampella G Damiati E NFKB1 regulates human NK cell maturation and effector functions. Clin Immunol (2017) 175:99–108. 10.1016/j.clim.2016.11.012 27923702

[22] Scaldaferri D Bosi A Fabbri M Pedrini E Inforzato A Valli R The human RNASET2 protein affects the polarization pattern of human macrophages in vitro. Immunol Lett (2018) 203:102–11. 10.1016/j.imlet.2018.09.005 30218741

[23] Baranzini N Pedrini E Girardello R Tettamanti G de Eguileor M Taramelli R Human recombinant RNASET2-induced inflammatory response and connective tissue remodeling in the medicinal leech. Cell Tissue Res (2017) 368(2):337–51. 10.1007/s00441-016-2557-9 28070637

[24] Zhao J Chen AX Gartrell RD Silverman AM Aparicio L Chu T Immune and genomic correlates of response to anti-PD-1 immunotherapy in glioblastoma. Nat Med (2019) 25(3):462–9. 10.1038/s41591-019-0349-y PMC6810613 30742119

[25] Darmanis S Sloan SA Croote D Mignardi M Chernikova S Samghababi P Single-cell RNA-seq analysis of infiltrating neoplastic cells at the migrating front of human glioblastoma. Cell Rep (2017) 21(5):1399–410. 10.1016/j.celrep.2017.10.030 PMC5810554 29091775

[26] Yuan J Levitin HM Frattini V Bush EC Boyett DM Samanamud J Single-cell transcriptome analysis of lineage diversity in high-grade glioma. Genome Med (2018) 10(1):1–15. 10.1186/s13073-018-0567-9 30041684 PMC6058390

[27] Amankulor NM Kim Y Arora S Kargl J Szulzewsky F Hanke M Mutant IDH1 regulates the tumor-associated immune system in gliomas. Genes Dev (2017) 31(8):774–86. 10.1101/gad.294991.116 PMC5435890 28465358

[28] Tirosh I Suvà MLJN-o Dissecting human gliomas by single-cell RNA sequencing. Neuro Oncol (2018) 20(1):37–43. 10.1093/neuonc/nox126 29016805 PMC5761500

