# Supplementary material for: Case Report: Single-Cell Transcriptomic Analysis of an Anaplastic Oligodendroglioma Post Immunotherapy
Source: Front Oncol. 2021 Jan 14;10:601452. doi: 10.3389/fonc.2020.601452 (PMC7841290; doi:10.3389/fonc.2020.601452)
Supplement: Supplementary file 1 [file DataSheet_1.pdf]

Supplementary Material

Supplementary Figures

Supplementary Figure 1 Dot plots showing the percentage and average expression of each cytolytic gene used to calculate the cytolytic score in T cells and NK cells, comparing between the enhancing and non-enhancing regions.

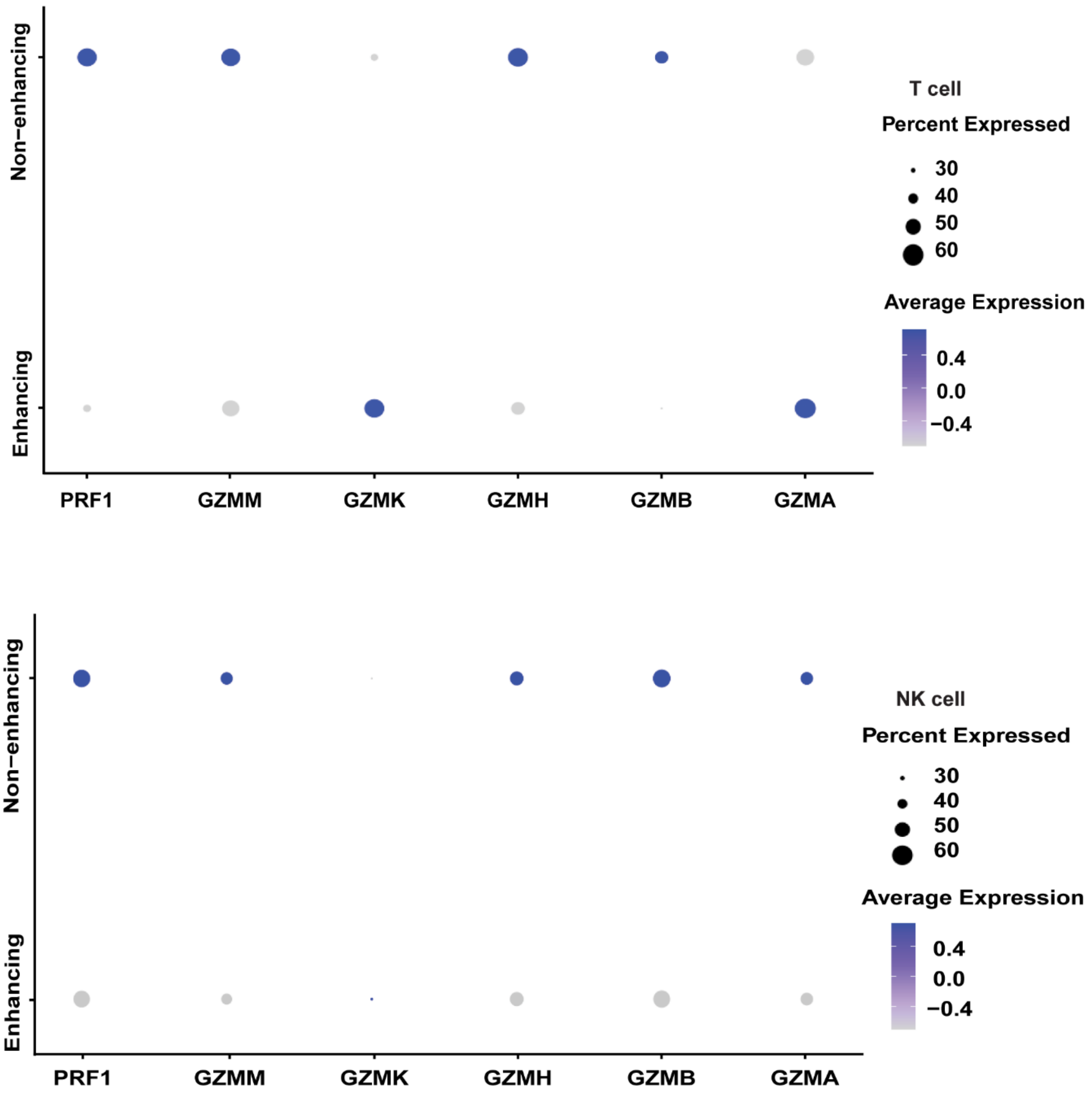

Supplementary Figure 2 Heatmap of significant genes shown in red (adj. p-value < 0.001 and fold change > 1.5) that are upregulated in the non-enhancing samples.

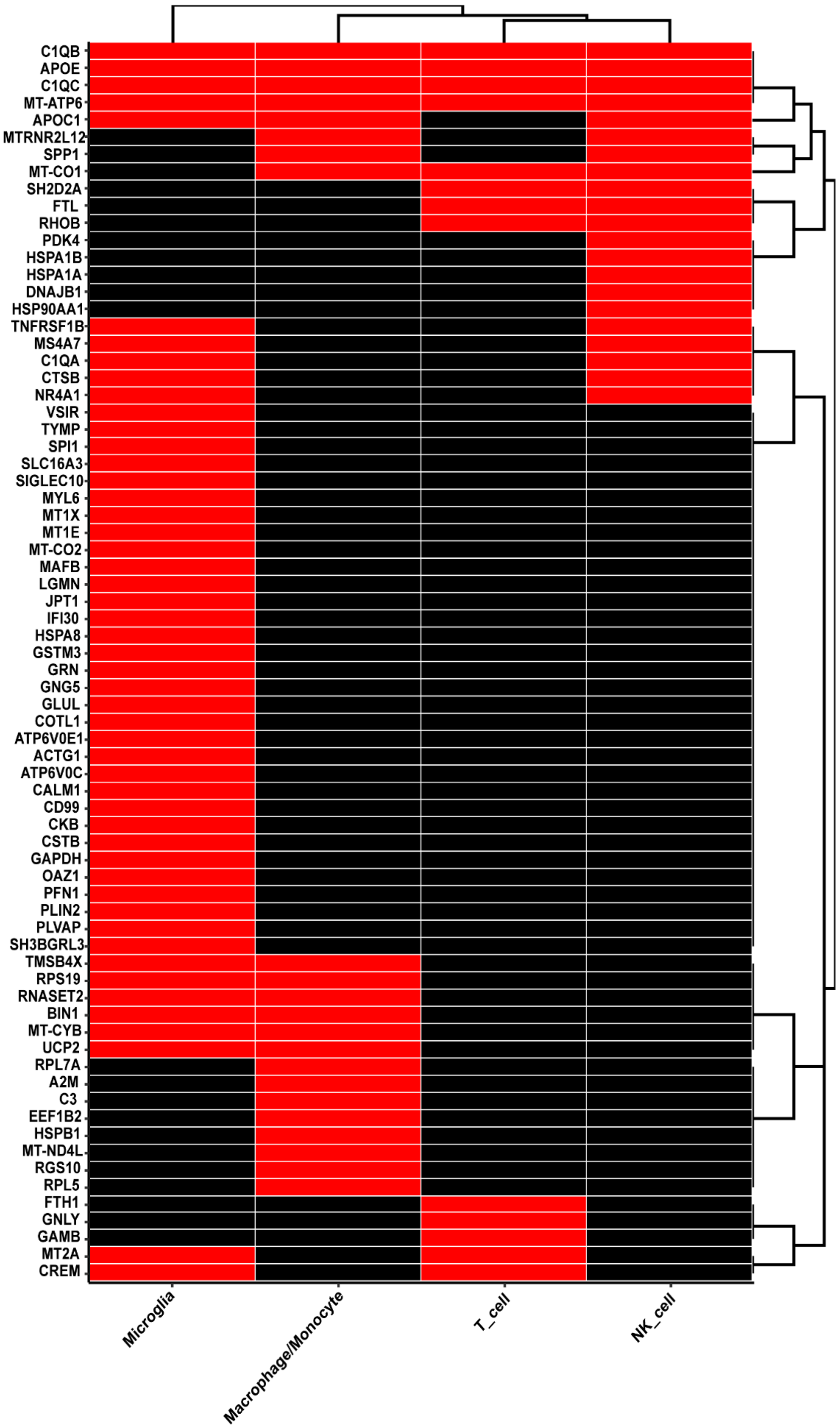

C1QB
APOE
C1QC
MT-ATP6
APOC1
MTRNR2L12
SPP1
MT-CO1
SH2D2A
FTL
RHOB
PDK4
HSPA1B
HSPA1A
DNAJB1
HSP90AA1
TNFRSF1B
MS4A7
C1QA
CTSB
NR4A1
VSIR
TYMP
SPI1
SLC16A3
SIGLEC10
MYL6
MT1X
MT1E
MT-CO2
MAFB
LGMN
JPT1
IFI30
HSPA8
GSTM3
GRN
GNG5
GLUL
COTL1
ATP6V0E1
ACTG1
ATP6V0C
CALM1
CD99
CKB
CSTB
GAPDH
OAZ1
PFN1
PLIN2
PLVAP
SH3BGRL3
TMSB4X
RPS19
RNASET2
BIN1
MT-CYB
UCP2
RPL7A
A2M
C3
EEF1B2
HSPB1
MT-ND4L
RGS10
RPL5
FTH1
GNLY
GAMB
MT2A
CREM
Microglia
Macrophage/Monocyte
T_cell
NK_cell

Supplementary Figure 3 Heatmap of significant genes shown in red (adj. p-value < 0.001 and fold change > 1.5) that are upregulated in the enhancing samples.

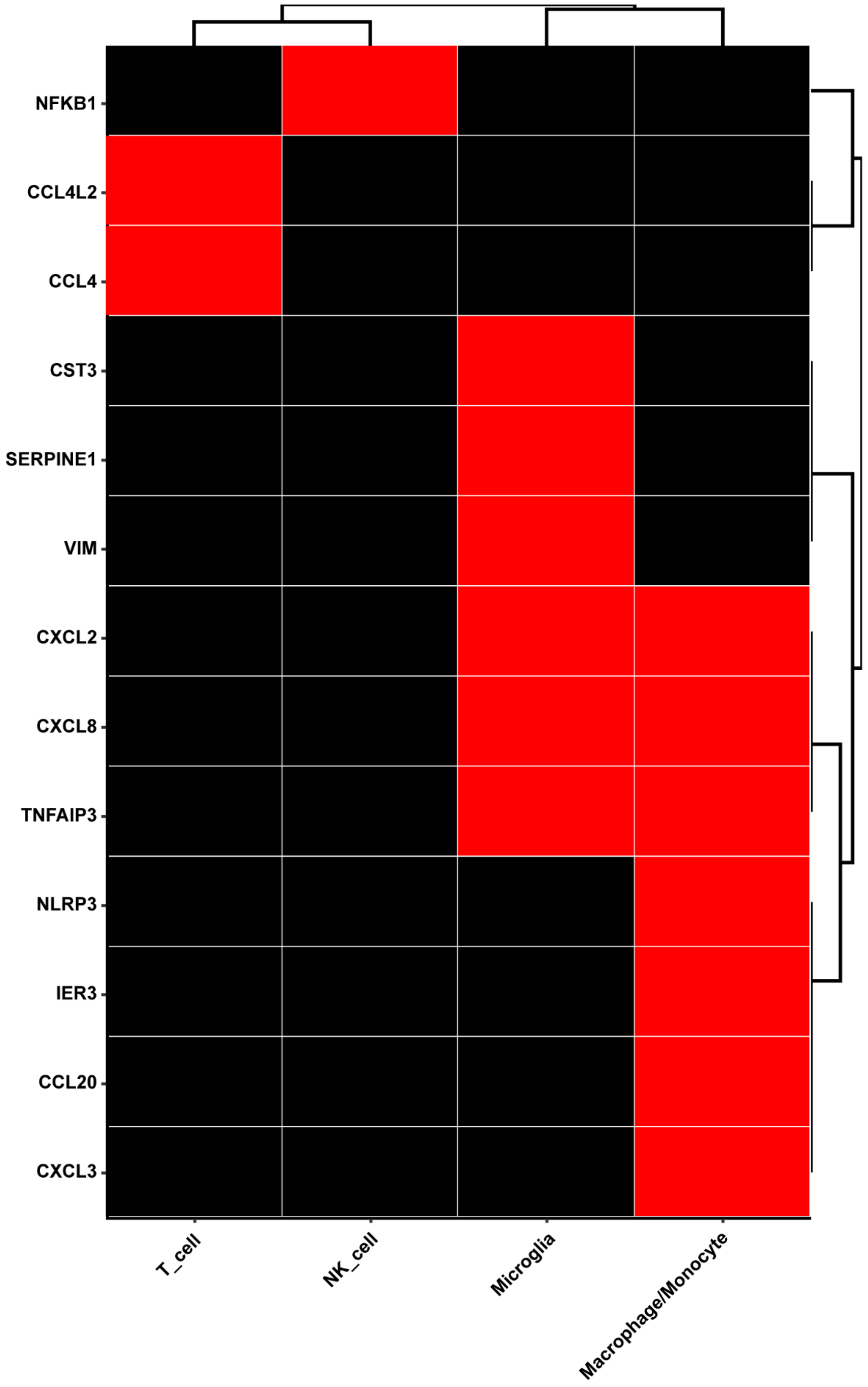
NFKB1
CCL4L2
CCL4
CST3
SERPINE1
VIM
CXCL2
CXCL8
TNFAIP3
NLRP3
IER3
CCL20
CXCL3
T_cell
NK_cell
Microglia
Macrophage/Monocyte

Supplementary Figure 4 Treatment outcome.

T1 post-contrast MRI images before the initiation of nivolumab and ipilimumab combined treatment (top panel), after two cycles of therapy (middle panel), and after 4 cycles of therapy (bottom panel), demonstrating the durable response.

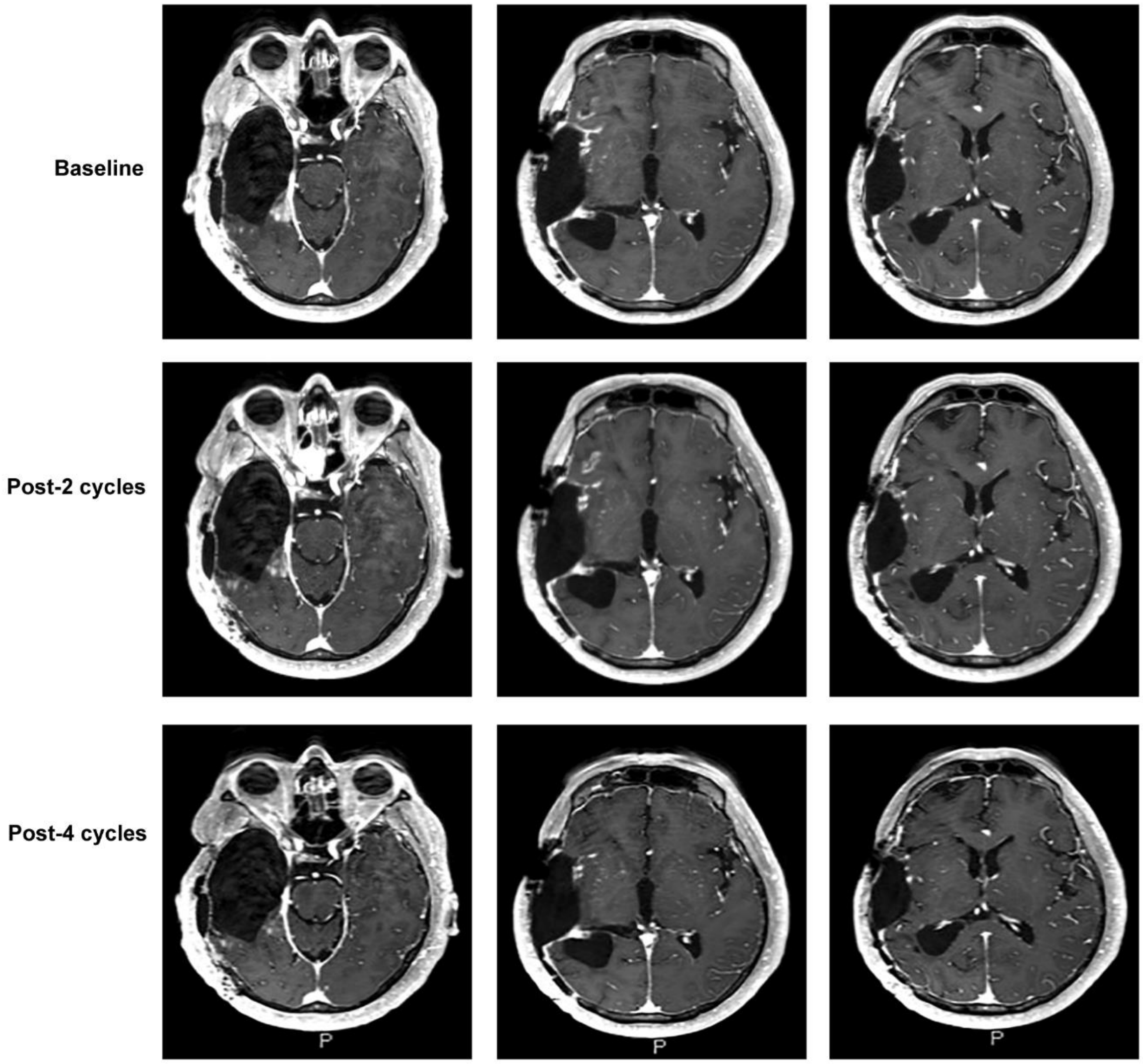

Methods and Materials

Tissue dissociation and processing

The enhancing and non-enhancing tissues from the operating room were transferred to the lab on ice immediately after the resection was performed. The samples were digested into single cells by using the Tumor Dissociation Kit (130-095-929, Miltenyi Biotech, Gladbach, Germany) or Liberase (5401119001, Sigma-Aldrich, St Louis, MO) for further single-cell analysis. Larger pieces were filtered out using a 70-µm filter. The samples were then treated with RBC lysis buffer (00-4333-57, eBioscience Invitrogen) to reduce the number of contaminating RBCs. Following RBC depletion, the dead cell removal protocol (130-090-101, Miltenyi Biotech) was conducted to enrich for cells with higher viability. The samples were washed twice with cold PBS to remove contaminating mRNA, and the sample concentration and cell viability were measured using a LunaFL fluorescent cell counter (Logos Biosystems, Inc). We chose the two samples with the highest viability from the enhancing and non-enhancing lesions for further sequencing analysis.

Library preparation and sequencing

Single cell suspensions from the samples were captured using the 10x Genomics 5' v1.0 Single Cell Chemistry according to manufacturer's instructions for partitioning and barcoding at a target recovery of 6,000 single cells per lane. Following RT-PCR and cDNA amplification, subsequent library construction and quality control were performed following the manufacturer's user guide. Sequencing for the two samples was performed by the CCR Genomics Core using the NextSeq 500/550 platform. Run parameters include 8bp index read, 28bp read 1, and 98bp read 2 for cDNA insert identification.

Data processing and analysis

Generation of FASTQ files and processing of data was performed using the cellranger (version 3.1.0) pipeline (10X Genomics). The read count per cell was above 50,000 and sequenced reads were aligned to the Human GRCH38 reference genome (refdata-cellranger-GRCh38- 3.0.0).

FASTQ files for each sample was aligned to the hg38 genome and empty barcodes were filtered with Cellranger V3 using the count function (1). Genes that have expression in less than 0.1 percent of total number of cells were then removed. Barcode filtration for low quality cells was done using outliers based on standard distribution of data (median +/- 3 deviations) for high percentage of mitochondria, low number of genes and low number of cells. Individual sample normalization was done using SCTransform through the Seurat V3 wrapper. Doublets were detected using DoubletFinder V2 with a doublet estimate of 3% and Individual cell annotations using SingleR package with the HPCA (Human Primary Cell Atlas) (2). Clustering, dimension reduction and sample integration was performed using the Seurat V3 Package. Clustering was performed using the Smart local moving (SLM) (3) and a resolution of 0.8 and projected using Uniform Manifold Approximation and Projection (UMAP) (4). Samples were integrated using the Integrate Data function in Seurat using 3000 anchor genes and default parameters. Average gene expression per cluster was calculated and clusters were annotated using SingleR with the HPCA dataset (5). Microglia cluster was further separated out from macrophages by using TMEM119, which was reported as a microglia specific marker (6). And oligodendroglioma was identified by using AUCell with enrichment dataset obtained from a previous report (7, 8). Reclustering was performed, subsetting the data and returning the clustering at a resolution of 0.6 using the top 20 principal components. Cytolytic scores were calculated by using the geometric mean of six cytolytic-related genes (PRF1, GZMM, GZMK, GZMH, GZMB and GZMA) as previous described (9).

Statistical analysis

Differential expression was performed using the FindMarkers function in Seurat with the option of using MAST for method which uses a hurdle model for handling the zero inflated single cell data (2). Genes were differentially expressed with fold change of 1.5 and false discovery rate adjusted p-value of 0.001. The fold change of cytolytic scores of T cells and NK cells was used in the comparison between enhancing and non-enhancing lesions. Significance of the differences between cytolytic score in enhancing versus non-enhancing lesion was analyzed by Wilcoxon test.

References

1. Stuart T, Butler A, Hoffman P, Hafemeister C, Papalexi E, Mauck III WM, et al. Comprehensive integration of single-cell data. *Cell.* (2019) 177(7):1888-902. e21. doi: 10.1016/j.cell.2019.05.031
2. Finak G, McDavid A, Yajima M, Deng J, Gersuk V, Shalek AK, et al. MAST: a flexible statistical framework for assessing transcriptional changes and characterizing heterogeneity in single-cell RNA sequencing data. *Genome Biol.* (2015) 16(1):1-13. doi: 10.1186/s13059-015-0844-5
3. Waltman L, Van Eck NJJTEpjB. A smart local moving algorithm for large-scale modularity-based community detection. *Eur Phys J B.* (2013) 86(11):471.
4. McInnes L, Healy J, Melville JJapa. Umap: Uniform manifold approximation and projection for dimension reduction. *ArXiv.* (2018) 1802.03426.
5. Aran D, Looney AP, Liu L, Wu E, Fong V, Hsu A, et al. Reference-based analysis of lung single-cell sequencing reveals a transitional profibrotic macrophage. *Nat Commun.* (2019) 20(2):163-72. doi: 10.1038/s41467-018-03751-6
6. Satoh Ji, Kino Y, Asahina N, Takitani M, Miyoshi J, Ishida T, et al. TMEM119 marks a subset of microglia in the human brain. *Neuropathology.* (2016) 36(1):39-49. doi: 10.1111/neup.12235
7. Aibar S, González-Blas CB, Moerman T, Imrichova H, Hulselmans G, Rambow F, et al. SCENIC: single-cell regulatory network inference and clustering. *Nat Methods.* (2017) 14(11):1083-6. doi:10.1038/nmeth.4463
8. Venteicher AS, Tirosh I, Hebert C, Yizhak K, Neftel C, Filbin MG, et al. Decoupling genetics, lineages, and microenvironment in IDH-mutant gliomas by single-cell RNA-seq. *Science.* (2017) 355(6332). doi: 10.1126/science.aai8478
9. Wei JS, Kuznetsov IB, Zhang S, Song YK, Asgharzadeh S, Sindiri S, et al. Clinically relevant cytotoxic immune cell signatures and clonal expansion of T-cell receptors in high-risk MYCN-not-amplified human neuroblastoma. *Clin Cancer Res.* (2018) 24(22):5673-84. doi: 10.1158/1078-0432.CCR-18-0599
